# Second International Diagnostic Accuracy Study for the Serological Detection of West Nile Virus Infection

**DOI:** 10.1371/journal.pntd.0002184

**Published:** 2013-04-25

**Authors:** Andrea Sanchini, Oliver Donoso-Mantke, Anna Papa, Vittorio Sambri, Anette Teichmann, Matthias Niedrig

**Affiliations:** 1 Centre for Biological Threats and Special Pathogens 1 (ZBS1) - Highly Pathogenic Viruses, Robert Koch-Institut, Nordufer, Berlin, Germany; 2 European Public Health Microbiology Training Programme (EUPHEM), European Centre for Disease Prevention and Control (ECDC), Stockholm, Sweden; 3 Department of Microbiology, National Reference Centre for Arboviruses, Medical School, Aristotle University of Thessaloniki, Thessaloniki, Greece; 4 Operative Unit of Clinical Microbiology, Regional Reference Centre for Microbiological Emergencies, S. Orsola-Malpighi University Hospital, Bologna, Italy; Johns Hopkins Bloomberg School of Public Health, United States of America

## Abstract

**Background:**

In recent decades, sporadic cases and outbreaks in humans of West Nile virus (WNV) infection have increased. Serological diagnosis of WNV infection can be performed by enzyme-linked immunosorbent assay (ELISA), immunofluorescence assay (IFA) neutralization test (NT) and by hemagglutination-inhibition assay. The aim of this study is to collect updated information regarding the performance accuracy of WNV serological diagnostics.

**Methodology/Principal findings:**

In 2011, the European Network for the Diagnostics of Imported Viral Diseases-Collaborative Laboratory Response Network (ENIVD-CLRN) organized the second external quality assurance (EQA) study for the serological diagnosis of WNV infection. A serum panel of 13 samples (included sera reactive against WNV, plus specificity and negative controls) was sent to 48 laboratories involved in WNV diagnostics. Forty-seven of 48 laboratories from 30 countries participated in the study. Eight laboratories achieved 100% of concurrent and correct results. The main obstacle in other laboratories to achieving similar performances was the cross-reactivity of antibodies amongst heterologous flaviviruses. No differences were observed in performances of in-house and commercial test used by the laboratories. IFA was significantly more specific compared to ELISA in detecting IgG antibodies. The overall analytical sensitivity and specificity of diagnostic tests for IgM detection were 50% and 95%, respectively. In comparison, the overall sensitivity and specificity of diagnostic tests for IgG detection were 86% and 69%, respectively.

**Conclusions/Significance:**

This EQA study demonstrates that there is still need to improve serological tests for WNV diagnosis. The low sensitivity of IgM detection suggests that there is a risk of overlooking WNV acute infections, whereas the low specificity for IgG detection demonstrates a high level of cross-reactivity with heterologous flaviviruses.

## Author Summary

West Nile virus (WNV) is mantained in the environment in a cycle between mosquitoes and birds. The virus has been isolated on almost all the continents, and several migratory bird species are primarily responsible for virus spread and dispersal. Humans acquire the infection through WNV-infected mosquito bites. Although most infected humans remain symptoms-free, in a minority of cases (especially in the elderly or immunocompromised patients) the infection can develop into a neuroinvasive form causing life-threatening encephalitis and threatening meningitis. Diagnosis of WNV is based primarily on serological tests, i.e. the detection of the virus-specific antibodies in human serum. Our aim was to collect updated information regarding the performance accuracy of WNV serological diagnostic tests used by laboratories involved in WNV diagnostics, in order to identify the strengths and weaknesses of diagnostic techniques in each laboratory. The performance of diagnostic tests varied among the laboratories, indicating that there is still a need to improve test procedures and to harmonize protocols.

## Introduction

West Nile virus (WNV) is a mosquito-transmitted flavivirus of the family *Flaviviridae* [1]. It is maintained in a cycle between birds and mosquitoes mostly belonging to the *Culex* genus [2]. *Ochlerotatus*, *Culiseta*, and *Aedes* mosquitoes are also competent vectors [2]. Besides horses and humans several other mammals are dead-end hosts of WNV [1], [2], [3].

About 80% of humans infected with WNV develop no or only very mild symptoms. In about 20% of the cases patients develop more severe symptoms such as fever, myalgia and lymphadenopathy. Furthermore, in small proportion of cases the infection progresses to life-threatening neuroinvasive forms characterized by meningitis, encephalitis and/or flaccid paralysis [1], [4]. The risk of developing lethal forms is increased in the elderly or in immunocompromised patients [1], [4].

WNV is the most widely spread flavivirus in temperate areas: it has been isolated in parts of Europe, Middle East, Africa, Asia, America and Australia, and migratory birds are responsible for the dispersal of the virus [5], [6], [7]. WNV is also capable of causing outbreaks of neuroinvasive infections, as demonstrated during outbreaks in Romania in 1996 (about 800 cases), in Greece in 2010–2012 (more than 500 cases, still ongoing), several outbreaks in the USA from 1999 to 2012, with over 15000 cases of neuroinvasive infections and about 1500 deaths and the recently confirmed WNV cases in Tunisia, in the Balkans and in Italy [8], [9], [10], [11], [12].

Both serological and nucleic acid-based tests are available for the diagnosis of WNV infections, but due to the short period of low viremia in humans, serological tests that detect virus-specific antibodies are more reliable [1], [13], [14]. Following infection with WNV, IgM antibodies are produced and can be detected within 4–7 days after exposure and may persist for about one year, while IgG antibodies can be reliably detected from day 8 after infection [15], [16].

There are several types of serological tests routinely used for WNV diagnostics: enzyme-linked immunosorbent assay (ELISA), immunofluorescence assay (IFA), neutralization test (NT) and the hemagglutination-inhibition assay. Commercial kits are available, but several laboratories have also developed their own in-house tests [1], [13], [17].

A major issue in WNV diagnostics is cross-reactivity with antibodies against heterologous flaviviruses, e.g. dengue virus (DENV), Japanese encephalitis virus (JEV), tick-borne encephalitis virus (TBEV) or yellow fever virus, which is especially true for IgG antibodies [18], [19]. NT is considered the most specific technique, but it is laborious, time-consuming and it can be performed only in BSL-3 laboratories, while ELISA is rapid, reproducible and cost-effective [1], [16]. In 2005, the European Network for the Diagnostics of Imported Viral Diseases-Collaborative Laboratory Response Network (ENIVD-CLRN) organized the first external quality assurance (EQA) study for WNV serological diagnostics to assess the performances of laboratories involved in WNV diagnostics [18]. The study revealed that the performance of diagnostic tests varies amongst laboratories and that there is need to improve them.

The aim of our study was therefore to update information on performance accuracy of WNV serological diagnostic tests used by expert laboratories through the organization of a second EQA study.

## Materials and Methods

### Participants and recruitment

Forty-eight laboratories involved in WNV serological diagnostics were invited to participate in this second EQA study. The study was organized by the ENIVD-CLRN network. The selection of the invited laboratories was based on the register of ENIVD-CLRN members as well as on their contributions to the literature relevant to this topic. The participation in the study was open and free of charge and included publication of the results in a comparative and anonymous manner.

The following 47 laboratories participated in the study:

Albania: Institute Of Public Health, Tirana. Argentina: Instituto de Virología “. J.M. Vanella”, Córdoba. Austria: Universität Wien, Wien. Belgium: Institute of Tropical Medicine, Antwerpen. Brazil: Instituto Evandro Chagas, Ananindeua. Bulgaria: National Center of Infectious and Parasitic Diseases, Sofia. Costa Rica: CNRV Inciensa, Cartago. Cuba: Institute for Tropical Medicine “Pedro Kourí”, Havana City. France 1: IRBA-IMTSSA Unité de Virologie, Marseille. France 2: LNR/LCR West Nile UMR1161, Maisons-Alfort. Germany 1: EUROIMMUN AG, Lübeck. Germany 2: Friedrich-Loeffler-Institut, Greifswald - Insel Riems. Germany 3: Institut für Mikrobiologie der Bundeswehr Zentralbereich Diagnostik, München. Germany 4: Institut für virologie, Hannover. Germany 5: Niedersächsisches Landesuntersuchungsamt, Hannover. Greece: Aristotle University of Thessaloniki, Thessaloniki. Iran: Pasteur Institute of Iran, Tehran. Italy 1: Amedeo Hospital, Torino. Italy 2: Istituto Superiore di Sanità, Rome. Italy 3: Lazzaro Spallanzani, Rome. Italy 4: Molecular Biology Section Army Medical and Veterinary Research Center, Rome. Italy 5: Istituto G. Caporale, Teramo. Latvia: Infectology Center of Latvia, Riga. Mexico: Lab. de Virus Hemorrágicos. Depto. Enfermedades Emergentes y Urgencias. Norway: Norwegian Institute of Public Health, Oslo. Portugal: National Institute of Health, Águas de Moura. Republic of Macedonia: Institute of Public Health, Skopje. Romania: Cantacuzino Institute, Bucharest. Russia: Central Research Institute of Epidemiology, Moscow. Saudi Arabia 1: Jeddah Regional Laboratory, Jeddah. Saudi Arabia 2: King Abdulaziz University Hospital, Jeddah. Senegal: Institut Pasteur, Dakar. Slovenia: University of Ljubljana, Ljubljana. South Africa 1: NHLS, Bloemfontein. South Africa 2: National Institute for Communicable Diseases, Johannesburg. Spain 1: Instituto de Salud Carlos III, Majadahonda. Spain 2: Clinic i Provincial de Barcelona, Barcelona. Spain 3: Investigadora Campus de Bellaterra, Barcelona. Sweden: Swedish Institute for Infectious disease control, Solna. Switzerland 1 : Spiez Laboratory, Spiez. Switzerland 2: Zentrum für Labormedizin, St Gallen. Switzerland 3: University Hospital of Geneva, Geneva. Turkey: National Public Health Agency, Ankara. The Netherlands 1: Erasmus MC, Rotterdam. The Netherlands 2: National Institute of Public Health and the Environment (RIVM), Bilthoven. U.K. 1: Health Protection Agency, London. U.K. 2: Animal Health and Veterinary Laboratories Agency, New Haw Surrey.

### Preparation and distribution of test samples

The preparation and distribution of the panels were carried out as previously described for the first EQA study on WNV diagnostics [18]. The instructions provided to the participants were also the same as for the previous EQA [18]. The test panel consisted of 13 different sera, including sera reactive against WNV as positive controls, sera reactive against heterologous flaviviruses as specificity controls and negative control sera.

The exact composition of the test panel was:

- 4 serial dilution of WNV BM140375 IgM/IgG positive serum (1∶1, 1∶2, 1∶4, 1∶8)
- one WNV BM141170 IgM negative/IgG positive serum
- one WNV WM(67/11) IgM low positive/IgG positive serum
- one WNV Greece (genetic lineage II) IgM low positive/IgG positive serum
- one DENV IgM negative/IgG positive serum
- one JEV IgM negative/IgG positive serum
- one TBEV IgM negative/IgG positive serum
- one Usutu virus (USUV) IgM negative/IgG positive serum
- 2 negative controls: one provided by EUROIMMUN (containing rheumatoid factor) and one in house negative control.

The DENV and the WNV plasma sera were obtained from plasmapherese centres from US and Costa Rica and were purchased from SeraCare Life Sciences, Milford, MA, USA. For TBEV and JEV the sera came from vaccinees, while for USUV the sample was provided by reference laboratories of ENIVD-CLRN network routine diagnostics.

### Ethics statement

All subjects provided informed oral consent. All samples taken from the collections were anonymized.

### Evaluation of participants' results

Two criteria were selected to evaluate the proficiency of each laboratory: 1) laboratories had to identify the seven WNV-positive serum samples irrespective of differentiation between IgM and IgG, i.e. if at least one of the test gave a positive result 1 point was assigned, and 2) the four serum samples containing cross-reactive antibodies to the heterologous flaviviruses (DENV, JEV, TBEV, USUV) and the two negative controls should not give a positive result and/or should be recognized as being unspecific. Equivocal or borderline results with the six non-WNV serum samples were interpreted as negative. False positive and false negative results were evaluated as incorrect and attributed a score of 0 points. The maximum score for each laboratory is 13 points (indicated as 100%), indicating that all diagnostic results were correct.

For each of the 13 serum samples, the score was assigned using identical criteria, allowing the percentage of laboratories giving correct results for each specific serum to be determined.

In order to be consistent and to make the results comparable, scoring criteria identical to those used during the first EQA study for the serological diagnosis of WNV infection were used [18]. The performances of the diagnostic tests with regard to IgM and IgG results were considered separately in order to give additional information concerning the quality of the laboratory diagnostics. Data were collected using Microsoft Excel (Microsoft Corp., Bellingham, WA, USA) and analysed using SPSS 14.0 for Windows. Results with respect to categorized variables were analysed by the chi-square test. A p-value<0.05 was considered to be statistically significant.

## Results and Discussion

### Participation to the study

Forty seven of 48 invited laboratories participated in the study (98% response rate). A total of 30 countries were involved, including 21 from Europe, 5 from America, 2 from Asia and 2 from Africa (see materials and methods section). A total of 51 tested panels were received for IgM and/or IgG detection because four laboratories sent two tested panels using both ELISA and IFA or NT test.

### Overall proficiency of the participants

Tables 1, 2 and 3 summarize the results obtained using ELISA, IFA and NT as detection method, respectively, and are sorted by percentage of correct results for each laboratory.

**Table 1:** Results of the EQA study for laboratories performing ELISA for the serological detection of West Nile virus.

	Samples																										
	#12,WNV		#5,WNV		#7,WNV		#6,WNV		#1,WNV		#3,WNV		#8,WNV														
	BM140375		BM140375		BM140375		BM140375		BM141170		WM67/11		(Greece)		#9,DENV		#4,JEV		#14,TBEV		#11,USUV						
	1∶1		1∶2		1∶4		1∶8		1∶2		1∶2		1∶2		1∶2		1∶2		1∶2		1∶2		#2,Neg		#13,Neg		
Lab	IgM	IgG	IgM	IgG	IgM	IgG	IgM	IgG	IgM	IgG	IgM	IgG	IgM	IgG	IgM	IgG	IgM	IgG	IgM	IgG	IgM	IgG	IgM	IgG	IgM	IgG	Sc
No.	(+)	(+)	(+)	(+)	(+)	(+)	(+)	(+)	(−)	(+)	(+)	(+)	(+)	(+)	(−)	(+)	(−)	(+)	(−)	(+)	(−)	(+)	(−)	(−)	(−)	(−)	%
32^H^	+	+*	+	+	+	−	+	−	−	+*	+*	+*	−	+	−	+*	−	−	−	−	−	−*	−	−	−	−	100
19^F^	+	+	+	+	e*	+	−	+*	+	+	+	+	−	+*	−	+*	−	−	−	e*	−	e	−	+*	−	−	100
31^B^	+	+*	+	+*	+	+*	+	+*	+	+*	+	+*	+	+*	+*	+*	−	+*	−	+*	−	+*	−	+*	−	+*	100
29b^F^	+	+*	+	+*	+	+*	−	+*	+	+*	+	+*	−	+*	−	+*	−	+*	−	+*	−	+*	−	+*	−	−	100
38^P^	+	+	e	+*	−	+*	−	+*	−	+*	+	+	−	+*	−	+*	−	−	−	−	−	−	−	+*	−	−	100
14^E^	+	+	+	+	−	+	−	e*	+	+	+	+	−	+	e	+*	−	−	−	−*	−	−	−	−	−	−	92
3^F^	+	+*	+	+*	+	+*	−	+*	+	+*	+	+*	−	+*	−*	+*	−	−	−	+*	−	−	−	+*	−	+	92
40^F^	+	+	+	+	e	+	−	+	+	+	+	+	−	+	+	+	−	−	−	−	−	e	−	−	−	−	92
23^F^	+	+	−	+	+	+	+	+	+	+	+	+	−	+	−	+	−	+*	−	+	−	+	−	+	−	+*	92
7^E^	+	+	+	+	−	+	+	+	+	+	+	+	−	−	−	+	−	−	−	−	−	−	−	−	−	−	85
36b^F^	+	+	+	+	+	+	−	+	+	+	+	+	−	+	−	+	−	−	−	e	−	e	−	+	−	−	85
16a^E^	+	+	+	+	−	+	−	e	−	+	+	+	−	+	−	+	−	−	−	−	−	−	−	−	−	−	85
6^H^	+	−	+	−	+	−	+	−	+	+	+	+	+	−	+	+	−	−	+	−	−	−	−	−	−	−	85
2^E^	e	+	e	+	e	+	e	+	−	+	+	+	−	+	−	+	−	−	−	+	−	−	−	−	−	−	85
15^E^	+	−	+	−	e	−	−	−	−	+	+	−	−	+	−	+*	−	−	−	−	−	−	−	−	−	−	85
45^F^	−	+	−	+	−	e	−	+	+	+	+	+	−	+	+	+	−	−	−	−	−	−	−	−	−	−	85
37^H^	+	+	+	+	e	−	e	−	+	+	+	+	+	+	−	−	−	−	−	−	e	+	−	−	−	−	77
20^P^	+	+	e	+	−	e	−	e	e	+	+	+	−	−	−	+*	−	−	−	−*	−	−	−	−	−	−	77
39^F^	+	nd	+	nd	e	nd	−	nd	+	nd	+	nd	−	nd	−	nd	−	nd	−	nd	−	nd	−	nd	−	nd	77
47^E^	+	+	+	+	e	e	−	−	−	+	+	+	−	+	−	+	−	−	−	−	−	−	−	−	−	−	77
8^S^	+	+	+	+	+	+	+	+	+	+	+	+	+	+	+	+	−	+	−	+	−	−	−	+	−	−	69
13^F^	+	+	+	+	+	+	+	+	+	+	+	−	−	−	−	+	−	−	−	−	−	+	−	+	−	−	69
1^F^	+	+	+	+	+	+	−	+	+	+	+	+	−	+	−	+	−	−	−	+	−	+	−	+	−	−	69
25^F^	+	+	+	+	+	+	−	+	+	+	+	+	−	+	−	−	−	+	−	+	−	+	−	+	−	−	69
24^E^	+	nd	+	nd	e	nd	−	nd	−	nd	+	nd	−	nd	−	nd	−	nd	−	nd	−	nd	−	nd	−	nd	69
43^P^	+	nd	e	nd	−	nd	−	nd	e	nd	+	nd	−	nd	−	nd	−	nd	−	nd	−	nd	−	nd	−	nd	69
44^H^	+	+	−	+	−	+	−	+	−	+	+	+	−	+	−	+	−	+	−	−	−	+	−	+	−	−	69
4^F^	+	+	+	+	+	+	e	+	+	+	+	+	−	+	−	+	−	+	−	+	−	+	−	+	−	−	62
18^F^	+	+	+	+	+	+	−	+	+	+	+	+	−	+	−	+	−	+	−	+	−	+	−	+	−	−	62
30^F^	+	+	+	+	+	+	+	+	+	+	+	+	−	+	+	+	−	+	−	+	−	+	−	+	−	−	62
26a^F^	+	+	+	+	e	+	−	+	+	+	+	+	−	+	+	+	−	+	−	+	−	+	−	+	−	−	62
27^P^	+	+	−	+	−	+	−	+	−	+	+	+	−	+	−	+	−	+	−	+	−	+	−	+	−	−	62
12^T^	+	+	+	+	+	+	+	+	+	+	+	+	+	+	+	+	+	+	+	+	+	+	+	+	+	+	54
33^H^	−	nd	−	nd	−	nd	−	nd	−	nd	+	nd	−	nd	−	nd	−	nd	−	nd	−	nd	−	nd	−	nd	54
46^H^	−	−	−	−	−	−	−	−	+	+	−	+	−	−	−	+	−	−	−	−	−	−	−	−	−	−	54
Sc %	94		91		71		69		94		100		77		43		74		66		66		63		94		

WNV: West Nile virus; DENV: dengue virus; JEV; Japanese encephalitis virus; TBEV: tick-borne encephalitis virus; USUV: Usutu virus; Neg: negative control; Sc: Score; IgM: Immunoglobulin M; IgG: Immunoglobulin G; nd: not done;

* : reactivity also with heterologous flaviviruses;

S : commercial ELISA InBios;

H : ELISA in-house test;

E : commercial ELISA Euroimmun;

F commercial ELISA Focus;

B : commercial ELISA Bioscreen;

P : commercial ELISA Panbio;

T : commercial ELISA IDVET.

**Table 2:** Results of the EQA study for laboratories performing IFA for the serological detection of West Nile virus.

	Samples																										
	#12,WNV		#5,WNV		#7,WNV		#6,WNV		#1,WNV		#3,WNV		#8,WNV														
	BM140375		BM140375		BM140375		BM140375		BM141170		WM67/11		(Greece)		#9,DENV		#4,JEV		#14,TBEV		#11,USUV						
	1∶1		1∶2		1∶4		1∶8		1∶2		1∶2		1∶2		1∶2		1∶2		1∶2		1∶2		#2,Neg		#13,Neg		
Lab	IgM	IgG	IgM	IgG	IgM	IgG	IgM	IgG	IgM	IgG	IgM	IgG	IgM	IgG	IgM	IgG	IgM	IgG	IgM	IgG	IgM	IgG	IgM	IgG	IgM	IgG	Sc
No.	(+)	(+)	(+)	(+)	(+)	(+)	(+)	(+)	(−)	(+)	(+)	(+)	(+)	(+)	(−)	(+)	(−)	(+)	(−)	(+)	(−)	(+)	(−)	(+)	(−)	(+)	%
17^E^	−	1∶320*	−	1∶80*	−	1∶20*	−	1∶20*	−	1∶320*	1∶160	1∶160*	−	1∶40*	−	1∶2560*	−	−*	−	−*	−	−*	−	1∶10*	−	−	100
21^E^	1∶40	1∶160	1∶20	1∶80	−	1∶20*	−	1∶20*	1∶20	1∶320*	1∶80	1∶160	−	1∶20	−	1∶80*	−	+*	−	1∶10*	−	1∶10	−	1∶10*	−	−	92
29a^H^	−	1∶256*	−	1∶128*	−	1∶64*	−	1∶32*	−	1∶1024*	1∶128	1∶1024*	−	1∶64*	−	1∶2048*	−	−*	−	1∶32*	−	+*	−	1∶16*	−	−	92
35^E^	+	+	+	+	+	+	−	e*	−	+	+	+	−	+	−	+	−	−	−	−	−	−	−	−	−	−	85
34^E^	+	+	+	+	−	−*	−	−*	+	+	+	+	−	+	−	−*	−	−*	−	−*	−	−*	−	−*	−	−	85
16b^E^	+	+	+	+	−	−*	−	−*	+	+	+	+	−	+	−	−*	−	−*	−	−*	−	−*	−	−*	−	−	85
11^E^	+	+	+	+	−	+	−	−	+	+	+	+	−	−	−	+*	−	−	−	−	−	−	−	−	−	−	85
28^H^	+	+	−	+	−	+	−	+	−	+	+	+	−	+	−	+	−	−	−	−	+	−	−	−	−	−	85
9^E^	−	1∶100	−	1∶100*	−	1∶100	−	1∶32	−	1∶320	1∶10	1∶320*	−	1∶100	−	1∶3200	−	−	−	−	−	−	−	1∶10	−	−	85
36a^S^	−	1∶320	−	1∶320	−	1∶160	−	1∶80	−	1∶640	1∶160	1∶640	−	1∶160	−	1∶2560	−	−	−	1∶80	−	1∶40	−	1∶40	−	−	69
41^H^	−*	−	−	−	−	−	−	−	−	+	+	+	−	+	−	−	−	−	−	−	−	+	−	−	−	−	62
Sc %	91		91		73		55		100		100		91		55		100		91		64		82		100		

WNV: West Nile virus; DENV: dengue virus; JEV; Japanese encephalitis virus; TBEV: tick-borne encephalitis virus; USUV: Usutu virus; Neg: negative control; Sc: Score; IgM: Immunoglobulin M; IgG: Immunoglobulin G; nd: not done;

* : reactivity also with heterologous flaviviruses;

E : commercial IFA Euroimmun;

H : IFA in-house test;

S : commercial IFA Scimedx.

**Table 3:** Results of the EQA study for laboratories performing NT for the serological detection of West Nile virus.

	Samples													
	#12,WNV	#5,WNV	#7,WNV	#6,WNV	#1,WNV	#3,WNV	#8,WNV							
Lab	BM140375	BM140375	BM140375	BM140375	BM141170	WM67/11	(Greece)	#9,DENV	#4,JEV	#14,TBEV	#11,USUV			Sc
No.	1∶1	1∶2	1∶4	1∶8	1∶2	1∶2	1∶2	1∶2	1∶2	1∶2	1∶2	#2,Neg	#13,Neg	%
26b^H^	+	+	+	+	+	+	+	−	−	−	−	−	−	100
42^H^	+	+	+	+	+	+	+	−	−	−	−	−	−	100
22^H^	1∶10	1∶10	−	−	1∶40	1∶40	−	−	−	−	−	−	−	77
10^H^	1∶20	1∶20	−	−	1∶60	1∶60	−	−	−	−	−	−	−	77
5H2	−	−	−	−	1∶30	1∶15	1∶40	1∶20	−	−	−	−	−	62
Sc %	80	80	40	40	100	100	60	80	100	100	100	100	100	

WNV: West Nile virus; DENV: dengue virus; JEV; Japanese encephalitis virus; TBEV: tick-borne encephalitis virus; USUV: Usutu virus; Neg: negative control; Sc: Score; IgM: Immunoglobulin M; IgG: Immunoglobulin G; nd: not done;

H : NT in-house test;

H2 NT in-house test (with WNV lineage II strain Austria).

The most widely used serological diagnostic test was ELISA, performed in 35 of 51 tested panels (69%), followed by IFA (in 11 of 51 tested panels, 22%) and NT (in 5 of 51 tested panels, 9%). Four laboratories using ELISA detected only IgM antibodies and no IgG antibodies.

In 37 of the 51 tested panels (73%) a commercial test was used, whereas an in-house test was used in the remaining 14 tested panes (27%).

According to the criteria given, heterogeneous scores were observed among the in-house and commercial tests used by the laboratories. Nevertheless, the scoring for in-house tests was the same as for commercial tests, ranging overall from 54 to 100%. In accordance with the first WNV EQA and as well as other EQA studies for the serological detection of DENV and hantavirus infections, there were no statistically significant differences in scores between laboratories using commercial or in-house assays [18], [19], [20], [21]. Interestingly, several laboratories using the same commercial kit but obtained different results (e.g. the panels 19 and 29 used ELISA kit “F” and gained 100% scores, and panels 4, 18, 26 and 30 also used ELISA kit “F” but gained 62% scores) as observed in the first EQA study [18]. This may highlight the need for some laboratories to perform correctly the test. However, in the panels 19 and 29 additional tests were performed with the negative controls which permitted to identify the false positive as heterologous flaviviruses (marked as +* in the tables). This indicates that the performance of additional tests for flaviviruses may help in interpreting the results, especially if not so high or borderline antibody-titres have been obtained.

No significant differences have been observed in performances among the different continents and among WNV-free and WNV-endemic countries. In countries reporting several panels (Spain, Germany and Italy) some slight differences exists, due also to the different methods used.

The best scores were obtained in eight tested panels (32, 19, 31, 29b, 38, 17, 26b and 42) where 100% of the diagnostic results were correct (13 out of 13 points) (Tables 1, 2 and 3). Of these eight tested panels, ELISA was performed in five, IFA in one and NT in two. In the other tested panels, the percentage of correct results varied from 54 to 92% (from 7 to 12 of 13 points). The major impediment preventing other laboratories from reaching the maximum level of performance was the cross-reactions with antibodies specific for heterologous flaviviruses, giving a high proportion of false-positives, especially for IgG detection. This is particularly true for cross-reactivity with DENV antibodies (serum sample #9) where only 21 of the 47 tested panels for IgG (44%) reported a correct result. Regarding the heterologous flaviviruses, (JEV, TBEV and USUV), correct results were reported in 37, 34 and 31 of the 47 tested panels for IgG respectively (equating to 79%, 72% and 66%). A statistically significant difference exists between the proportion of correct results for DENV and for the other three flaviviruses (p<0.05). For the serum sample #2 (negative control) a correct result was reported in 36 of the 51 tested panels (71%). The high number of incorrect results with this negative control could be due to the presence of auto-antibodies that were reactive in the WNV serological tests. Serum sample #8 represents the genetic lineage II strain of WNV and was correctly detected in 40 of the 51 panels (78%). The WNV lineages I and II have about 30% of nucleotide divergence and showed antigenic variability, as observed in in cross neutralization analyses and monoclonal antibody binding assays [22], [23], [24], [25]. The antibody titre of the lineage II reactive serum was 1∶100 for IgM, 1∶1000 for IgG. Serum samples #3 and #1 (two WNV genetic lineage I strains) were correctly detected in 100% and 98% of the panels, respectively, giving the highest rate of correct results. Serum samples #12, #5, #7 and #6 were 4 serial dilutions of a WNV genetic lineage I strain, and results for these serum samples showed a decrease in sensitivity with increasing dilution (Tables 1, 2 and 3).

### Performances of the different serological tests

Considering only ELISA, 29 of 35 (83%) tested panels were obtained using a commercial test whereas 6 of 35 (17%) tested panels were obtained using an in-house test.

For IFA, 8 of 11 tested panels were obtained using a commercial test (73%) whereas 3 of 11 tested panels (27%) were obtained using an in-house test. All NT were in-house tests.

Scores ranged from 54 to 100% in tested panels using ELISA, from 62 to 100% for tested panels using IFA and from 62 to 100% for tested panels using NT. No statistically significant difference was observed in the scores of the three different serological tests.

Considering the scores related to each serum sample, it is possible to draw conclusions about the sensitivity and specificity of the different tests, particularly when comparing ELISA and IFA results, as there were only 5 laboratories performing NT.

The evaluation of sensitivity (capacity to detect true positives) involves the serum samples positive for WNV (serum samples #12, #5, #7, #6, #1, #3, #8 in tables 1, 2 and 3). For ELISA the sensitivity was 54% and 87% with respect to IgM and IgG detection, while for IFA the sensitivity was 45% and 86% with respect to IgM and IgG detection. One difference in sensitivity between ELISA and IFA is observed for the detection of the WNV lineage II. IFA seems to be more sensitive than ELISA for the detection of WNV lineage II (91 and 77% respectively), although, as no statistically significant difference was observed, this is only a tendency.

The evaluation of specificity (capacity to detect the true negatives) involves the serum samples negative for WNV (serum samples #9, #4, #14, #11, #2, #13 in tables 1, 2 and 3). For ELISA the specificity was 94% and 64% with respect to IgM and IgG detection, while for IFA the specificity was 99% and 85% with respect to IgM and IgG detection The IFA was more specific than ELISA in detecting IgG antibodies (p-value<0.05).

Although only 5 laboratories performed NT, it is interesting that low sensitivity was observed even for the highest concentrations of the WNV serum (Table 3).

### Performances of the tests with respect to IgM and IgG detection

As the test cannot distinguish between IgM and IgG antibodies, NT is excluded from this analysis.

A result for IgM detection was reported for 46 tested panels. The percentage of IgM antibodies correctly detected by the serological tests was 71%, with a sensitivity of 50% and a specificity of 95%. The low sensitivity for IgM detection can be explained mainly by the low detection of IgM antibodies of the WNV lineage II (serum sample #8): correct results were reported only in 5 of 46 tested panels.

As previously described in other EQA studies, such a low sensitivity for IgM detection leads to a risk that acute WNV infections can be overlooked [18], [21], and this can be especially dangerous for infections with the lineage II, which has been increasingly isolated and involved in outbreaks in recent years [26].

A result for IgG detection was reported for 42 tested panels. The percentage of IgG correctly detected by the serological tests was 78%, with an overall sensitivity of 86% and a specificity of 69%. The low specificity for IgG detection can be explained mainly by the cross-reactivity of the test with antibodies of heterologous flaviviruses, especially DENV (serum sample #9): correct results were reported only in 8 of 42 tested panels. As previously reported, cross-reactivity is a well-known problem for serological assays especially among flaviviruses [17], [18], [19], [20].

### Comparison between first and second EQA study of WNV serological diagnostics

In this second study, the number of participating laboratories was almost double that of the first study (47 and 27, respectively) [18]. In addition, coverage has increased, with 30 countries being involved in this second study compared to 20 in the first [18].

The number of serum samples included in the panel for the second study was increased from 10 to 13. The serum sample positive for WNV strain belonging to the genetic lineage II, the four serum samples in serial dilution and serum sample positive for JEV and USUV were included in this second study. There was no improvement in the number of laboratories that achived the 100% score in this second study compared to the first [18]. This could be due to difficulties in detecting the WNV lineage II and/or in detecting the higher dilutions of the WNV serum and/or the high level of cross-reactivity with DENV antibodies.

The percentage of IgM antibodies correctly detected in this study increased from 62% to 71% while the percentage of IgG correctly detected decreased from 88% to 78%.

A total of 16 laboratories participated in both studies. Among these, five laboratories increased their score, ten laboratories decreased their score and one laboratory had the same score.

However, as the number and the nature of serum samples were different in these two studies, comparisons of performances between the two studies need to be considered carefully.

Finally, as previously described, uneven performances and results have been obtained among laboratories using the same test [18].

The results of this second EQA study on WNV serological diagnostics demonstrate that there is still need to improve tests (either in-house or commercial), and to improve the test procedures used by laboratories.

Contrasting test performances were observed with respect to IgM detection (low sensitivity), or IgG detection (low specificity). Reliable assays for IgM detection are crucial for the diagnosis of acute or recent infections in humans and therefore their development is of first priority. Increasing of specificity for IgG detection is the second objective improving the diagnostics of WNV infection.

The results of an EQA study allow all participant laboratories to identify problems and to improve their performances, as well as to receive feedback via a final anonymized report and guidance upon request from the ENIVD-CLRN network. To improve diagnostic tests performances, for any positive results identified by ELISA or IFA, a second confirmatory more specific test should be applied, e.g. NT. Of remarks, in our study only five laboratories performed a NT test for WNV diagnosis. Moreover, due to the persistence of IgM antibodies [14], [15], a pair of samples should be tested for all suspected cases combined with IgG avidity test to distinguish among recent and past WNV infections [27].

Due to the cross-reactivity with heterologous flaviviruses, other diagnostic tests with heterologous flaviviruses should be performed to better identify false-positive results.

The broadening of the number of participants for this second EQA study compared to the first gave us a better overview of the strengths and weaknesses regarding the serological diagnosis of WNV infection. The increasing spread of WNV lineage II in Europe should be taken into account when establishing new diagnostic assays and evaluating performance in the future.
